# Translational Difficulties in Studying the TRPA1 Receptor

**DOI:** 10.3390/nu8120790

**Published:** 2016-12-03

**Authors:** Daniel Keszthelyi, Mark van Avesaat, Freddy J. Troost, Ad A. Masclee

**Affiliations:** Division of Gastroenterology-Hepatology, Department of Internal Medicine, Maastricht University Medical Center, 6202AZ Maastricht, The Netherlands; m.vanavesaat@maastrichtuniversity.nl (M.v.A.); f.troost@maastrichtuniversity.nl (F.J.T.); a.masclee@mumc.nl (A.A.M.)

Dear Editor,

We read with interest the article by Fothergill et al. [1] presenting data on the potential role of transient receptor potential ankyrin 1 (TRPA1) and the functional presence of this receptor in mouse intestine. The authors show that activation of the TRPA1 receptor by its agonists causes an increase in short-circuit currents. Such a phenomenon could offer a theoretical basis for enhancing nutrient handling efficiency. Interestingly, this effect was not related to either nerve activation nor serotonin release, as shown by additional tetrodotoxin and granisetron experiments, respectively.

Given the frequent use of flavouring substances in the food industry [2], as well as the accumulating evidence on the role of the chemo- and mechanosensory properties of TRPA1 channels [3,4], we have recently conducted a study in 12 healthy controls, in which 66 mg of the TRPA1 agonist cinnamaldehyde (Aldrich W228613, food grade; dose of approximately 0.7 mg/kg bodyweight, which equals the acceptable daily intake as per the recommendation of the Joint FAO/WHO Expert Committee on Food Additives) was infused into the duodenum over 30 min using a nasoduodenal catheter. The study employed placebo (saline) and capsaicine (transient receptor potential vanilloid 1-TRPV1-agonist) as comparators in a single-blind fashion and was partly reported elsewhere (van Avesaat et al. [5], clinical trials number NCT01667523). We hypothesized that stimulation of the TRPA1 receptor by cinnamaldehyde would cause an increase in small intestinal permeability (as measured by an oral sugar test) and serotonin release (as measured in duodenal biopsies and plasma). However, we found no significant changes in either of the parameters measured. In addition, on the contrary to the infusion of capsaicin, no subjective symptoms, such as fullness, pain or nausea were elicited.

The question therefore arises, why we were not able to reproduce the experimental findings of Fothergill et al. [1], but also others [6], in humans. An obvious explanation would be that the dose administered was insufficient to exert these effects. On the other hand, Fothergill et al. also point to previous discrepancies in human and animal data with regards to changes in transepithelial conductance [7]. It might very well be the case that in vitro, in vivo animal and human studies are difficult to extrapolate. This might particularly be the case for studies on TRPA1, for which only preliminary human data are available [8].

This question becomes particularly relevant as the presumed role for cinnamon supplements in a glycemic control has repeatedly been suggested but hitherto not been firmly established [9,10]. Further mechanistic studies in humans will be necessary to ascertain the exact effects of cinnamon and cinnamaldehyde in humans and whether any health benefit can be accounted for by TRPA1-mediated effects.
